# Mental health in the time of polycrisis: geopolitical determinants and modern psychiatry

**DOI:** 10.3389/fpsyt.2026.1813883

**Published:** 2026-04-24

**Authors:** Dinesh Bhugra, Michael Liebrenz, Alexander Smith

**Affiliations:** 1 Institute of Psychiatry, Psychology & Neuroscience, King’s College London, London, United Kingdom; 2 Department of Forensic Psychiatry, University of Bern, Bern, Switzerland

**Keywords:** climate change, conflict, geopolitical determinants, geopsychiatry, migration, polycrisis

## Abstract

Psychiatry is increasingly being practised in environments affected by geopolitical instabilities, including economic fragmentation, democratic backsliding, and widening inequities. The confluence of these phenomena contributes to what has been described as a contemporary polycrisis, encompassing synchronous disruptions that reinforce one another and threaten collective wellbeing. Nevertheless, psychiatric research and clinical work have generally remained oriented towards immediate determinants and risk factors, overlooking the macro-level political and institutional dynamics that can condition stressor exposure and mental health disparities. Amidst, interconnected crises, this paper advances geopsychiatry as a framework for understanding how distal geopolitical determinants translate into psychiatric vulnerabilities across communities and societies. Focussing on armed conflicts, climate change, and forced migration as emblematic domains of polycrisis, it highlights how these compounding phenomena are generating direct mental health burdens and may amplify harms via secondary pathways. Moreover, it contends that the psychiatric consequences of polycrisis are unlikely to be ameliorated through patient-centred interventions alone, but also require innovative approaches responsive to structural inequalities and material forces that transcend borders. In this context, work from geopsychiatry can offer important implications for modern psychiatry, highlighting a need for a more globally representative evidence base, potential clinical adaptations, and policy engagement that better attends to the geopolitical determinants of mental health.

## 1 Introduction

Amidst ongoing geopolitical upheaval, modern psychiatry is not insulated from the macro-level dynamics that are redrawing borders and weakening societal cohesion (1). In parts of the world, displacement and hunger have surpassed levels seldom witnessed, yet the proposed solutions from certain political actors have been to build walls and enact punitive anti-immigration policies (2, 3). Multilateral cooperation is splintering, not least for commitments to the World Health Organization and the United Nations (UN) Sustainable Development Goals, with the latter largely receding from view (4–6). Trade tariffs and financial uncertainty persist, even as wealth inexorably concentrates upwards (7). Compounding these pressures is a normalisation of unempathic leadership and performative cruelty, alongside the disintegration of democratic values and confidence in institutions, which have engendered existential concerns about civic instabilities and the spectre of catastrophic conflicts (8–10).

This coalescence of interlocking disruptions has been referred to as a polycrisis (*polycrise*), wherein singular crises amplify and perpetuate others, degrading the capacity (and the sociopolitical will) to respond (11, 12). Polycrisis entails simultaneous and cumulative tensions emerging throughout multiple spheres, undermining systemic resilience and exacerbating health disparities amongst vulnerable groups (6, 13–15). Although the polycrisis construct has been criticised for its analytical ambiguity and its tendency to centre Northern experiences of crisis (12, 16), it nonetheless captures something distinctive about current events that narrower theories may struggle to convey. Indeed, recent scholarship contends that polycrisis is inseparable from established trajectories of global inequalities and the uneven landscapes of adversity they have produced, which mainstream discourse has been slow to acknowledge (16–18).

Mental health is no exception, but psychiatric research and clinical practice are fundamentally patient-centred and have therefore tended to focus on personalised and community-based determinants (*e.g.*, biomarkers, genetic predispositions, critical life events, living conditions, etc.), underpinned by the biopsychosocial model. Recently, however, there has been growing attention towards the broader processes that threaten physical and mental wellbeing (19–22). Accordingly, geopolitical determinants of mental health have been described as governance-linked and transnational drivers, which are distinct from, but interact with, social and commercial determinants (19–22).

In an interconnected world, these may operate through security agendas, resource distribution, mobility, institutional functioning, and additional mechanisms, creating psychosocial burdens that resonate well beyond their origins (19–24). For instance, immediate, deleterious impacts can arise for people living in conflict zones, climate-affected settings, refugee camps, and detention facilities (5, 22–27); faraway effects often follow via displacement chains, family separation, legal precarity, and the algorithmically-driven transmission of graphic content and disinformation generated by Artificial Intelligence (AI) (5, 22–28). For contemporary psychiatry to address these challenges and better serve diversifying patient groups, both require explanation, and both require multidisciplinary, systems-level thinking, whilst remaining attentive to individual clinical encounters and exigencies.

Consequently, albeit a nascent subdiscipline, geopsychiatry has gained traction, seeking to interrogate these determinants through a psychopathological lens and complementing social determinants and other macro-level approaches to the structural drivers of mental health. Specifically, geopsychiatry examines how exposure and risk factors extend from transnational spheres to populations and communities (1, 5, 22–27). In applying biopsychosocial foundations to geopolitical realities, geopsychiatry illustrates how distal forces and policy decisions influence proximal stressors and lived experiences, aligned with principles of social justice and respect for human rights (1, 27). Concomitantly, geopsychiatry leverages insights from distinct epistemologies (*e.g*., anthropology, law, political science, sociology etc.) (1, 5, 22, 27). In doing so, it accentuates bilateral, cross-cultural exchanges over the unidirectional exporting of knowledge, which has long been a tension within global mental health (29, 30).

**Figure 1:**
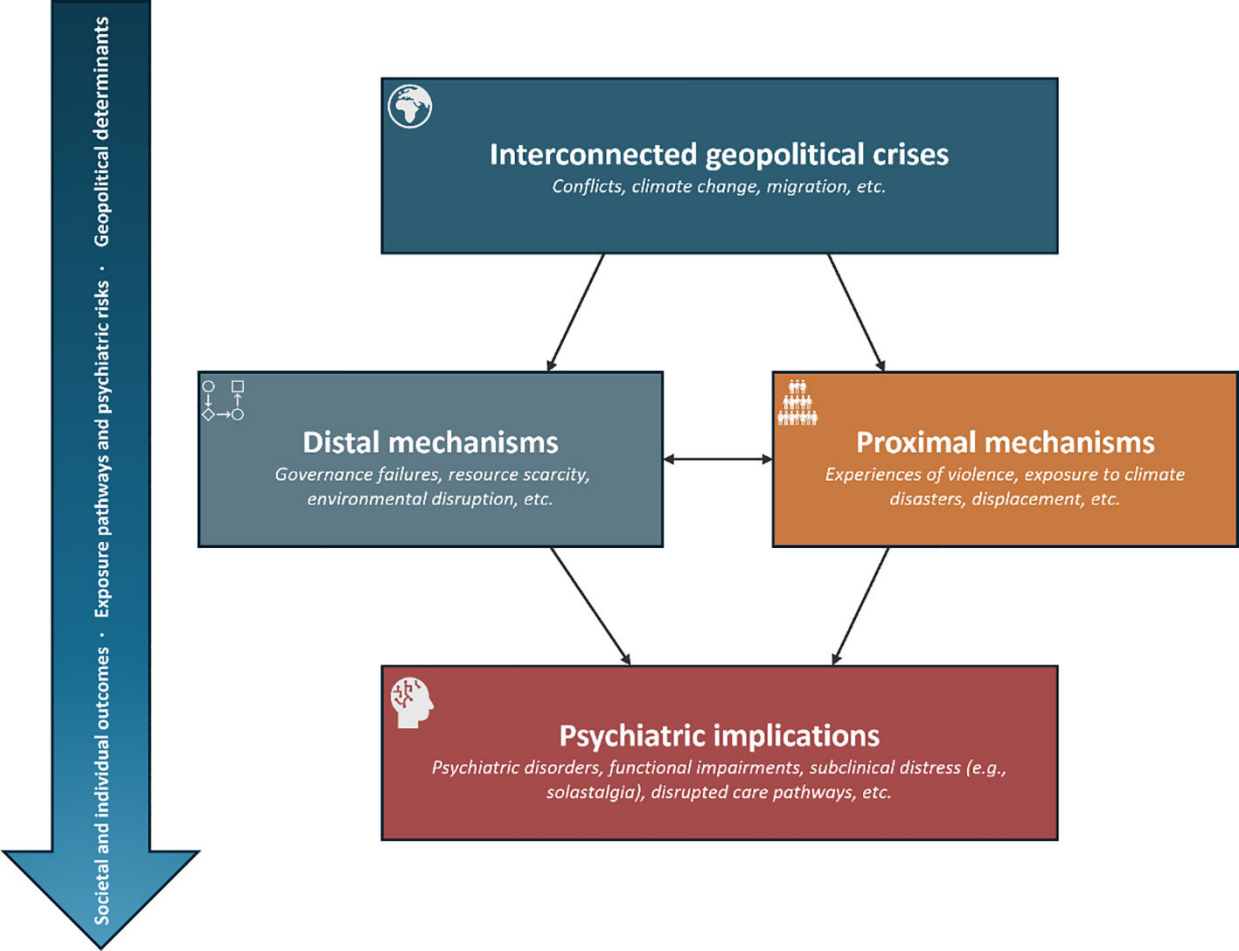
Geopolitical determinants and pathways to mental health outcomes.

Against this background, this paper draws on geopsychiatry as an organising framework for exploring how geopolitical determinants can shape psychiatric outcomes across three intersecting and intensifying phenomena, namely: armed conflicts, climate change, and migration. These domains are not treated as an exhaustive taxonomy of crises, but as emblematic and mutually reinforcing pathways through which contemporary geopolitical instability translates into psychiatric vulnerabilities at societal and individual levels (**Figure 1**).

Informed by a targeted, non-systematic narrative synthesis that prioritises foundational conceptual contributions, recent evidence syntheses, and illustrative data, this paper highlights psychiatric implications that may not be resolvable through patient-centred interventions alone (22–27). Thus, in an era of polycrisis, it concludes by affirming the importance and limitations of geopsychiatry as the psychiatric discipline confronts rapidly changing contexts of practice.

## 2 Geopolitical determinants and psychiatric outcomes

### 2.1 Armed conflicts

Armed conflicts represent the starkest sign of geopolitical breakdown and, by t measure, current events offer little reassurance. At the time of writing, there are more ongoing conflicts than at any juncture since the end of the Second World War, and the psychiatric impacts of this level of violence are both immediate and long-lasting (23, 25). Specifically, proximal conflict-related correlates include persistent exposure to critical life events, human rights violations, gendered abuse, bereavement, displacement, and damage to civic infrastructure that ordinarily serve as protective psychosocial factors (*e.g.*, housing, schooling, etc.) (31, 32).

Meta-analytic data suggests that around 22% of people living in warzones exhibit psychiatric symptoms, with rates of depression, anxiety, and post-traumatic stress disorder typically exceeding baseline comparisons (31, 32). However, these figures likely underestimate the real prevalence, given disruptions to routine epidemiological surveillance and the limitations of translating diagnostic criteria developed in peacetime (and largely Western paradigms) to environments pervaded by conflicts (19, 33). In this sense, the suffering of affected populations may well be obscured by the conditions that sustain it.

By definition, the destruction of medical infrastructure during conflict scenarios is itself a compounding mechanism, notwithstanding laudable initiatives to provide psychiatric care on the frontline (25, 34). As has recently transpired in the Democratic Republic of Congo, Myanmar, Sudan, regions in the Middle East, and elsewhere, the bombardment of healthcare facilities (including psychiatric hospitals), disruption to pharmaceutical supply chains, and forced physician migration exacerbate care needs (25, 35, 36). Notably, following the Syrian Civil War (2011-2024), over 70% of healthcare workers were killed or displaced, and only some hospitals remain fully operational as of 2025 (37). Meanwhile, in the Tigray War (2020-2022) in Ethiopia, healthcare provisions collapsed rapidly, with fewer than one-third of hospitals functional within six months (38). These are rarely incidental occurrences, but instead emanate from intentional military-strategic decisions and flagrant violations of human rights and international law (39).

In addition to these direct mental health effects, the indirect consequences of conflicts propagate in disparate contexts. Conflict-induced displacement can engender psychiatric burdens throughout entire migration trajectories and beyond into host communities, wherein legal precarity and discrimination may consolidate past adversities (24, 40). Vicarious exposure also represents a secondary pathway, with studies associating media consumption of war-related imagery, as well as fears of imminent conflict, with poorer mental health outcomes (41, 42). For example, research conducted in Egypt found that repeated viewing of conflict scenes from Gaza was associated with high levels of adolescent psychological distress (43). Separately, evidence on the transmission of trauma suggests that experiences of violence in one generation could plausibly influence psychopathological risks in the next, as emphasised in post-colonial frameworks (44).

More broadly, conflicts intersect with climate change and international systems in ways that epitomise the geopolitical linkages of the contemporary polycrisis (6, 11–18). Globally, military forces entail a significant carbon footprint, which, combined with environmental devastation from kinetic strikes, can further aggravate the psychosocial sequelae of warfare via climate disruption and food and resource insecurity (25, 45). As of March 2026, the Iran War exemplifies these dynamics, with monitoring agencies documenting widespread toxic contamination, marine pollution, and air quality issues arising from strikes on oil refineries and materiel, affecting millions of civilians (46). Climate change also contributes to the incidence and escalation of armed conflicts, especially in lower-income countries, thereby producing interdependent feedback loops with enduring implications for mental health and institutional functioning (47).

Hence, from the perspective of geopsychiatry, conflicts are embedded within a wider matrix of harms, demanding greater attention to preventive strategies, governance failures, and structural inequities rooted in histories of disadvantage. Nonetheless, predominant framings of polycrisis may reproduce these asymmetries, having gained currency primarily through Global North narratives and elite policy discourse, something that geopsychiatry, with its onus on bilateral exchanges, seeks to redress (12, 16, 22).

### 2.2 Climate change

Amidst record-breaking global temperatures and increasingly frequent extreme weather events, climate change also poses distinct geopolitical challenges for modern psychiatry (26). Several drivers of climate change reflect decades of economic and policy inaction under neoliberal systems that have subordinated environmental regulation to growth imperatives, worsening psychosocial vulnerabilities worldwide (22, 48). Akin to conflicts, these can mechanistically arise via proximal and distal pathways, rendering isolated interventions insufficient to mitigate cumulative burdens (26, 49–53).

Whilst causality is difficult to substantiate, systematic reviews have highlighted numerous firsthand associations between higher ambient temperatures and the onset of psychiatric symptoms. This includes proneness to composite mental illness, suicidality, and inpatient admissions (52–54). Heat-related issues entail difficulties for service planning, with time-series analyses showing correlations between rising temperatures and psychiatric emergency presentations, notwithstanding divergent demographic patterns (52–54). Likewise, adverse weather events have been tied to accentuated trends in depression, anxiety, PTSD, schizophrenia, and other disorders (49–54).

The distribution of these burdens is inherently stratified, since many low-income countries bear disproportionate impacts from climate change, despite contributing least to its major causes (55). These inequities have increasingly been framed using climate justice and post-colonial theories, and are, in no small part, a product of a hegemonic economic order that has historically outsourced industrial costs onto those with the least power to resist them (56, 57). Notably, data from the UN indicates that the 45 “least developed countries” generate under 4% of total carbon emissions (58). Accordingly, this raises questions about the universality of the evidence-base on climate and psychiatry, which continues to be skewed towards high-income jurisdictions, thereby perpetuating epistemic imbalances that persist throughout global mental health literature (59, 60). Ultimately, such disparities merit awareness, lest the society’s most susceptible to climate injustices remain the least visible (55).

Alongside these direct outcomes, climate change can have secondary implications for mental wellbeing, many of which have geopolitical intersections. For example, eco-anxiety (*i.e.*, chronic worries about environmental changes), ecological grief (*i.e*., mourning for degraded ecosystems), and solastalgia (*i.e*., distress arising from environmental changes to one’s home surroundings) have emerged as subclinical phenomena; these have been identified in communities exposed to shifting climates, as well as those anticipating future harms (61–63). Evidence syntheses highlight how Indigenous communities are particularly affected, with land dispossession driven by mining, logging, and fossil fuel extraction amplifying mental health burdens across Australia, Brazil, Canada, Chile, Peru, and beyond (64).

Relatedly, a cross-national survey of children and young people reported widespread climate-related concerns and functional impairments, with psychological distress coinciding with negative appraisals of governmental policies (65). Conceptual work suggests that these reactions may be understandable (and potentially adaptive) (66). In turn, this blurs the boundaries between psychopathology and normative responses to existential crises, invoking questions about possible diagnostic inflation, as well as what constitutes prevention and effective care in these contexts (61, 63, 67). For example, advanced AI climate modelling can strengthen psychosocial preparedness, although its concentration in high-income nations risks deepening extant inequities (68).

Moreover, climate ruptures are compromising supply chains at the macro-level, leading to food insecurity and conceivable deficiencies in the availability of micronutrients that support neurodevelopment and cognitive functioning (51). Environmentally-induced migration can entail complex psychiatric consequences, inflected by fractured social relationships and the loss of connection to land, again resonating with histories of dispossession (56, 69, 70). Finally, as previously described, climate change is entangled with armed conflicts, and extant research (where available) suggests that environmentally-induced displacement exacerbates resource competition and psychiatric vulnerabilities across borders (25, 47, 71). As such, this has led to climate change being epitomised as a mental health “threat multiplier”, which signifies the geopolitical interdependencies that underpin both geopsychiatry and the contemporary polycrisis (24).

### 2.3 Forced migration

With over 120 million people forcibly displaced worldwide, migratory trends are mediated by geopolitical forces (*e.g.*, climate change, armed conflicts, persecution, etc.) and can entail substantial psychiatric implications, thereby constituting a key concern for geopsychiatry (23, 26, 41, 72). Indeed, psychological risk factors may transpire at every stage of the migration trajectory, including critical life events, human rights abuses, and exploitation before and during transit through to legal precarity, acculturation stressors, and discrimination experienced in host regions (41, 73). Accordingly, recent evidence syntheses have outlined a sizeable prevalence of psychiatric disorders in refugee and asylum seeker groups, which often cluster syndemically with broader health and social disparities (32, 73, 74).

Moreover, the mental health burden of forced migration has been shown to be cumulative and context-contingent, with patterns varying by time-since-displacement and resource availability within destination countries (75). Equally, longitudinal work on refugees highlights bidirectional relationships between psychiatric symptoms and psychosocial functioning, with distress degrading support networks and perceived social obligations intensifying complex psychological issues (76). Conversely, access to housing and employment and supportive resettlement schemes have been linked to attenuated psychiatric risks, underlining the modifiable (and preventable) nature of certain post-migration stressors (2, 77).

Policy decisions also generate indirect mental health consequences for displaced and host communities, especially where punitive approaches are prioritised (78). For instance, elevated psychiatric symptoms have been identified in immigration detention spanning Australia, the United Kingdom, the United States, Switzerland, and other countries (79). Child-parent separations due to detention and deportation have been linked to lasting developmental problems (80). Deportation can cultivate “psychological homelessness”, where individuals no longer feel they belong in their country of origin (81). This sense of displacement can be intensified in offshore detention facilities, a policy gaining traction in high-income countries, which has been shown to cause dehumanisation and moral injury, as articulated by detainees in Nauru (2, 82). Elsewhere, data from Germany indicates that state-sponsored repatriation programmes can engender heightened psychiatric vulnerabilities, in part due to concerns about reintegration (83).

These circumstances may create spillover effects beyond those directly involved; in the United States, citizens who knew someone who had been detained or deported reported higher rates of anxiety and depression (84). Divisive political rhetoric characterising migrants as threats, “invaders”, or “colonisers” might amplify these harms by legitimising discrimination and heightening hostility amongst host-community members, to the detriment of social cohesion (25, 85). These tensions are frequently reinforced by exclusionary practices that restrict legal entitlement, social protections, and accessible care (86).

At the time of writing, populist anti-immigration narratives are proliferating in high-income nations, which ironically host a relatively small proportion of displaced people worldwide (2, 19); 75% reside in low- and middle-income countries that together account for less than 20% of global economic output, often in temporary settlements (*e.g*., refugee camps) where prolonged stays have been associated with deteriorating mental health (87). In lower-income settings, the largescale inward migration of displaced populations further strains already limited public health systems and mental health infrastructure (25). In Lebanon, for example, Syrian refugees have been shown to have substantial mental health needs amidst documented gaps in service provision and treatment access (88).

## 3 The role of geopolitical determinants in modern psychiatric practice

Collectively, these interdependent pathways illustrate how conflicts, climate change, and displacement reinforce each other, leading to multifactorial consequences (6, 11–19, 21). In a globalised but fragmented geopolitical landscape, geopsychiatry provides a framework for distilling the broader factors influencing localised patterns of exposure and the availability of mental health care (1, 5, 22–27).

Whilst social determinants and public health frameworks have been transformative, they have tended to focus on national or sub-national factors, leaving geopolitical dynamics comparatively undertheorised (19, 22). Simultaneously, geopolitical determinants should not be prioritised in ways that eclipse local idioms of distress or overstate the explanatory reach of transnational forces in complex psychiatric presentations. At its extreme, this risks introducing “concept creep”, whereby an expanding geopolitical lens gradually subsumes an ever-wider range of adversities through psychopathological interpretations (89).

Nonetheless, the polycrisis constructs highlights the limits of narrowly focussed approaches, underlining the need to combine culturally adaptive and forward-thinking strategies that are explicit about the geopolitical determinants impinging on mental health and psychosocial functioning (1, 5, 22–27). Ultimately, a more diverse and globally inclusive evidence base is needed to inform scalable prevention and health promotion efforts, which better incorporate insights from underrepresented and crisis-affected areas (59, 60, 90, 91). Specifically, future investigations should prioritise neglected settings, like refugee camps and regions recurrently facing climate disasters (33, 47, 59, 60). Although security concerns and ethical considerations can preclude access to these environments, ameliorating entrenched epistemic imbalances will require novel methodologies and equitable partnerships (92).

Greater emphasis is warranted for qualitative and participatory designs that foreground contingent narratives of distress and resilience, rather than relying on epidemiological screening predicated on diagnostic classifications developed in comparatively stable (and primarily Western) jurisdictions (18, 33). This is critical for individuals who may be suffering but also for affected societies, given the possibilities of diagnostic misattribution in underserved and historically disadvantaged areas (30). Furthermore, research should continue to explore the causal interconnections between ongoing crises and their psychiatric sequalae, since these are difficult to isolate (*e.g*., the associations between climate disruption, displacement trajectories, and mental health) (71, 90).

Naturally, this work shares intersections with considerations about service planning changes, since mental health systems will increasingly need to adapt to turbulent geopolitical contexts and diversifying patient groups (6, 23). The bidirectionality and interdisciplinarity that define geopsychiatry can inform these discussions (5). In this regard, the environmental footprint of normative psychiatric care structures will likely eventually necessitate moves towards lower-carbon services (23, 93); here, community-based models with demonstrable efficacy in the Global South could serve as instructive examples, but would require extensive testing and adaptation (22, 93, 94). Likewise, interprofessional task-shifting and digital health solutions underpinned by AI could bolster the delivery of mental health and psychological first aid in conflict zones where formal psychiatric facilities have collapsed (95, 96).

These priorities (and realities) need to be embedded in training; for medical practitioners to understand the health effects of polycrisis, educational structures must evolve (6, 97, 98). Central to this should be dedicated resources on advocacy, cultural competence, critical thinking, systems theory, and geopolitical determinants, particularly as patient populations continue to diversify (23, 25, 99–102). However, currently, training standards and validated pedagogical approaches for many of these issues remain largely underdeveloped worldwide. Finally, these initiatives should be accompanied by sustained policy engagement to help drive meaningful change at population and systemic levels (14, 21). To that end, leaders skilled in diplomacy will be important for future international health policy and governance, capable of handling complex political demands and simultaneously strengthening interdisciplinary and cross-jurisdictional cooperation (15). Equally, from a practical perspective, policy-oriented tools have been created in geopsychiatry, including the CAPE Vulnerability Index, which uses 26 indicators to identify countries in need of foreign assistance (25).

That said, whether progress in these spheres is realistically achievable is uncertain, and the current geopolitical climate admittedly provides little cause for optimism, not least given the rise of anti-science and state-sponsored health misinformation (19, 103). Still, the urgency is proportional to the difficulty, and no single domain will be able to solve these challenges alone (including geopsychiatry), nor from a biopsychosocial perspective can geopolitical factors solely account for the full complexity of individual symptom presentations (25). Hence, for psychiatry to continue to respond to societal needs in an unstable epoch, it must engage with the intersecting crises that are undermining mental wellbeing across the world, and do so through cross-national and cross-disciplinary collaborations (1, 5, 15, 22–27).

## 4 Conclusion

In a period punctuated by synchronous crises, psychiatric practice is already contending with (and will increasingly confront) macro-level factors that are transcending national borders and detrimentally affecting mental wellbeing. Geopsychiatry offers instructive frameworks for understanding how conflicts, climate change, forced migration, and other geopolitical issues interact as combined drivers of psychiatric vulnerabilities. This perspective paper elucidated these dynamics through illustrative examples, highlighting several associated research, training, and policy needs, whilst acknowledging the conceptual limits of geopsychiatry as an emerging subdiscipline.

Psychiatry has long centred on viewing mental health and patient needs *in situ*, which must continue to be prioritised; however, this context is now unmistakably global, and may inexorably be bound up with considerations about social justice and human rights. As transnational instabilities and inequalities grow, so too does the case for an outward-looking and forward-thinking psychiatric discipline, attuned to the geopolitical determinants and material conditions underlying who suffers, who receives care, and, ultimately, who gets left behind.

## Data Availability

The original contributions presented in the study are included in the article/supplementary material, further inquiries can be directed to the corresponding author/s.
